# Motivational Interviewing Adapted to Group Setting for the Treatment of Relapse in the Behavioral Therapy of Obesity. A Clinical Audit

**DOI:** 10.3390/nu12123881

**Published:** 2020-12-18

**Authors:** Elena Centis, Maria L. Petroni, Veronica Ghirelli, Mattia Cioni, Paola Navacchia, Emilia Guberti, Giulio Marchesini

**Affiliations:** 1 Department of Medical and Surgical Sciences, IRCCS Policlinico Sant’Orsola-Malpighi, Alma Mater University of Bologna, Via Massarenti, 9, I-40138 Bologna, Italy; elena.centis@aosp.bo.it (E.C.); matialetizia.petroni@unibo.it (M.L.P.); 2 Local Health Unit, Department of Public Health, Food and Nutrition Service, Via Altura 3, I-40139 Bologna, Italy; p.navacchia@ausl.bologna.it (P.N.); emilia.guberti@gmail.com (E.G.); 3 Alma Mater University, Via Massarenti, 9, I-40138 Bologna, Italy; veronica.ghirelli@studio.unibo.it (V.G.); mattia_cioni@hotmail.com (M.C.)

**Keywords:** body weight control, obesity, secondary prevention, discrepancy, physical activity, self-efficacy, stage of change

## Abstract

Motivational interviewing (MI) is devised to change unhealthy behaviors by increasing motivation. We adapted MI to a group format for the treatment of relapse during the behavioral treatment of obesity and performed a clinical audit to evaluate its effectiveness in stopping weight regain. The program was structured in seven weekly sessions, plus a 6-month follow-up. Patients (*n* = 86) completed a questionnaire on motivation to change in both healthy diet and physical activity, and a self-reported measurement of calorie intake and physical activity at baseline, at program end and at 6-month follow-up. The attendance to the program was high, with only 13 patients (15%) not completing the program and 24% not attending the 6-month follow-up. By the end of follow up, the prevalence of patients in either precontemplation or contemplation was reduced from over 60% at enrollment to approximately 20%, whereas the sum of patients in action or maintenance stages was increased from 9.5% in healthy diet and 14% in physical activity to 39.7% and 41.3%, respectively. These changes translated into significant behavioral changes (mean calorie intake, −13%; total physical activity, +125%; sedentary time, −8%) and finally into reduced body weight ( −3%). We conclude that MI programs adapted for groups may be used to stop relapse in individuals following a behavioral intervention for obesity.

## 1. Introduction

The behavioral treatment of obesity is hindered by several difficulties, barriers and obstacles that put progressive weight loss and weight loss maintenance at risk. In several cases the amount of weight necessary to achieve the expected weight loss targets is exceedingly large, and the more challenging the targets, the higher the risk of treatment attrition [1]. In general, weight loss reaches its peak within 6 months from treatment start; this time period may be insufficient to achieve the desired targets and, in the absence of a weight maintenance program, 50% of patients return to their original weight within 5 years [2]. 

The high rates of attrition and weight regain prompted the development of innovative programs fostering motivation and adherence [3]; this is especially important in the setting of public health departments and public hospitals where resources are limited as compared to a disproportionally large potential audience. Motivational interviewing (MI) is a collaborative style of conversation aimed at strengthening motivation and commitment to change [4]. The basic assumption is that people already have reasons and the resources to change; the purpose of the MI is simply to guide individuals in a process of “internal exploration” so as to recognize and verbalize motivations for change and to become confident in their ability to achieve and maintain behavioral changes over time. The effectiveness of MI in the treatment of obesity has been extensively evaluated [5,6], either as a standalone intervention or as an adjunct to other approaches [7], in both the adult and the pediatric setting [8,9]. Although MI was initially described as an individual approach, several experiences of MI adapted to a group format are available at present [10] but mainly refer to addiction treatment. MI groups are defined as groups that use the MI spirit, processes and techniques to increase motivation for change, that foster healthy interaction among members and leaders, meeting in a shared physical space, to promote change [10].

MI is usually carried out during the first visit, on an individual basis. Following this approach, patients may be addressed to dietitian-managed nutritional counseling programs, or to more intense group programs of cognitive-behavioral therapy (CBT) [11]. The choice is made depending on the severity of obesity and on the results of appropriate questionnaires indicating a more severe distress and/or the presence of eating disorders. In order to strengthen motivation in our patients on long-term weight loss, we structured a formal MI program that was offered to patients who had started weight regain at various stages of their treatment program. The reasons for adding MI to standard CBT have been largely discussed [12]; the two strategies largely overlap also in their conceptual framework, with differences in patient–therapist interaction [4,13].

We designed a clinical audit for defining the role of our MI program in the context of obesity treatment for patients with obesity unable to adhere to weight loss maintenance strategies. This report aims to describe our MI program adapted to the group context and the clinical characteristics of psychological profile, behavioral parameters and body weight of enrolled patients, as well as treatment effects. 

## 2. Materials and Methods

### 2.1. Patients

We report data on 86 patients, enrolled in the MI group program in the course of the years 2018–2019. Their sociodemographic, psychological and clinical data are reported in Table 1. Eligibility criteria included age below 70, weight regain after the end of the weight loss program, associated with decreased attention to behavioral strategies, but continuing participation in follow-up. The program was proposed to all cases fitting these criteria, but only 55% of cases agreed, because of job or family constraints, preventing weekly attendance to MI groups. 

The study was carried out as part of a parallel clinical activity carried out both in the Department of Medical and Surgical Sciences, Alma Mater University, Bologna, and the Department of Public Health, Local Health Unit, Bologna. 

### 2.2. Motivational Interviewing Program

Group MI consists of four phases: (i) engaging the group; (ii) exploring perspectives; (iii) broadening perspectives and (iv) moving into action [10]. The first phase (first meeting) is aimed at building the group and a collaborative atmosphere to encourage the conversation about change. The group leader and the members introduce themselves in a welcoming and empathetic atmosphere that facilitates interaction. The general program, the objectives and approaches, as well as the expectations, are presented and discussed by the team leader and the participants, respectively. The second phase focuses on exploring participants’ current condition, which includes lifestyle, habits, ambivalence about change, etc. Two heuristic models are presented: the stage of change according to Prochaska and Di Clemente [14] and the ready–willing–able model, proposed by Miller and Rollnick [4]. The aim is to share tools that allow members to reflect on their motivation for change. In this phase, the concept of ambivalence is introduced and, through the use of the decisional balance, the leader accompanies participants in an attempt to recognize the problems and difficulties produced by their behavior. The third phase—broadening perspectives—aims at evoking and reinforcing the motivation for change. Through the use of specific strategies, such as decisional balance, exploring values, exploring importance and confidence and change success stories, group members are stimulated to an internal exploration process to facilitate greater awareness of any inconsistency between personal values and present behaviors, as well as of the internal and external resources that can make change possible. The last phase—moving into action—is aimed at planning change through the identification of specific, measurable and feasible objectives and customized strategies for achieving them.

At this stage, the participants are ready to recognize and meet the obstacles that jeopardize the achievement of their objectives and to overcome them. This phase ends with a final meeting, which represents a moment of particular importance. The leader encourages members to declare how they intend to continue, to express what they liked in the group and the members, what they “take-home” and it is emphasized that the end of group meetings does not represent the end of their personal process of change.

The attendance to the program was high, with only 13 patients (15% of cases) not completing the program. Sixty-five cases also attended the 6-month follow-up. All psychometric and behavioral tests were repeated three times: at enrollment, at the end of the program and at 6-month follow-up. 

### 2.3. Questionnaires 

Motivation to change was tested using the EMME-3 questionnaire for healthy diet and habitual physical activity [15], derived from a previously validated tool for individuals with alcohol problems [16]. The questionnaire consists of two parallel sets of instruments (for diet and physical activity, respectively): (a) an 18-item questionnaire (MAC 2) on a Likert scale from 0 (totally false) to 6 (totally true); (b) a set of 6 visual analogue scales (VAS) from 0 to 100. A third part of the test, containing 9 brief descriptions (PORTRAITS) of imaginary people, confirmatory of the same motivational components of the MAC 2 questionnaire, was not used in this setting. 

The answers to the different questions are summed up to evaluate motivation to change according to the Prochaska model of stages of change [17] (precontemplation, contemplation, determination, action, maintenance) using 10 statements, two for each stage. The scores of the 5 stages provide a graphic summary of the stage-of-change profile. The area with the highest score was considered as the prevalent stage of change.

The remaining eight questions of MAC 2R, combined with the VAS responses, provide scores on discrepancy, self-efficacy, importance, temptation, readiness-to-change and stabilization-of-change. Discrepancy refers to the contradiction between what a person is or behaves like and what one aims to be or to behave like, related to personal “image of self”, values, goals and expectations [18]. When habitual diet and low physical activity are considered as threats to health, individuals perceive a need for change, but they may be unable to make the desired changes [19]. Discrepancy (also named internal fracture) reflects concern and dissatisfaction with the present situation (need for change) and the perceived importance of change (desire for change). Self-efficacy, as defined by Bandura [20], is the perceived confidence in attaining and maintaining the predefined goals of change. It has been extensively evaluated in the area of alcohol abstinence as a key factor in dealing with high-risk situations and reaching the desired targets [21,22]. Importance and temptation are defined as the importance attributed to the new lifestyle and the attractive value of the old lifestyle; finally, readiness-to-change and stabilization-of-change offer a summary assessment of the stage of change.

The questionnaires showed good internal consistency with theoretical assumptions, reliability and concurrent validity in a large study of 431 subjects, most of whom were overweight or obese [15].

### 2.4. Calorie Intake and Physical Activity

The measurement of calorie intake was measured by an in-house-developed questionnaire (QMV (Quanto Mangio Veramente?)—How much do I really eat?) [23,24]. The questionnaire provides a semi-quantitative estimation of the average daily calorie intake, validated in an extensive analysis of patients seeking medical treatment for obesity [23]. Total calorie intake is computed as the sum of calories derived from frequency of intake and calories of the average portion of 19 items included in our daily diet. In order to have an indication of the intake of the different nutrients (carbohydrates, proteins and lipids), the items of the questionnaire were also grouped on the basis of their prevalent composition (cereals, bread, pasta, sweets, cakes/ice creams, potatoes and fruits for carbohydrates; meat for protein; pork meat, oil and cheese for lipids). 

Habitual physical activity was measured by the International Physical Activity Questionnaire (IPAQ) [25]. The value of weekly energy expenditure (in MET-hour/week) is derived from the computation of frequency (weekdays), duration (minutes per bouts) and intensity (light/moderate/vigorous) of sports or other habits (e.g., walking, cycling) involving energy expenditure; the time spent sedentary was also recorded. Participants who did not report any physical activities were defined as inactive. 

### 2.5. Anthropometric and Biochemical Measurements

Height and weight were measured on a standard scale at half centimeter and kilogram. Waist circumference was measured with a tape at the midpoint between the lower rib limit and the superior iliac spine. Obesity was diagnosed in the presence of body mass index (BMI, weight (in kg)/height^2^ (in m)) ≥30 kg/m^2^.

### 2.6. Statistical Analysis

All data were implemented on a personal computer and analyzed using StatView 5.0™ program (ABACUS Concepts, Inc., Berkeley, CA, USA). Initially, analysis of covariance failed to indicate significant differences in principal baseline characteristics between the various MI groups (not reported in detail) and the different groups were merged. All parameters were tested for normal distribution and descriptive statistics (mean ± standard deviation or median (interquartile range) for non-normally distributed characteristics) was carried out on the whole dataset, as well as on data split according to gender. Differences in individual parameters between groups were analyzed by Student’s t test or Mann–Whitney test, whenever appropriate. The chi-square test was used to compare prevalence between groups. The time trends of individual variables, as well as the differences between values measured at baseline and those reported at the end of the MI program (approximately 2–3 months later) and those obtained at 6-month follow-up, were calculated per protocol and compared by paired t test and Wilcoxon rank test.

*p* values <0.05 were considered statistically significant.

## 3. Results

The large majority of cases were women (76.9%), in the age range 32–75 (median 55.5) and median BMI at entry of 34.7 kg/m^2^ (interquartile range, 6.3). Only 22% of cases were in the overweight range (Table 1). Their body weight was approximately 50% increased, compared with weight at early adulthood, and very close to their lifetime maximum body weight, particularly in women. They reported an extremely variable average calorie intake, scarce physical activity and a lot of sedentary time (particularly in men, 523 ± 220 min/day vs. 388 ± 170 in women; *p* = 0.034). Ten percent of patients were defined as physically inactive.

**Figure 1:**
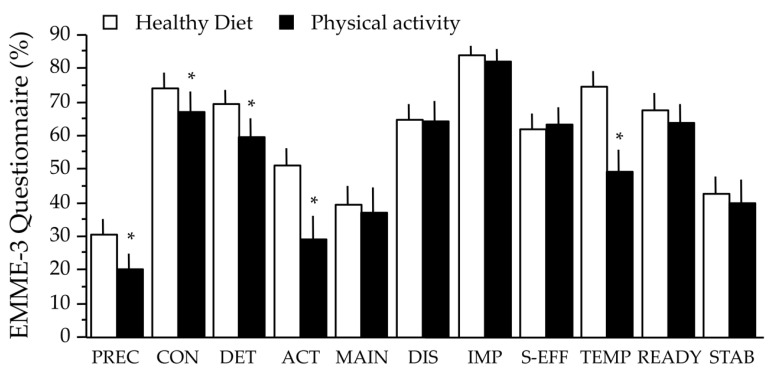
Scores of the EMME-3 questionnaire for healthy diet and physical activity at baseline. Data are expressed as mean ± SE. The asterisks indicate a significant difference between diet and physical activity (*p* < 0.05, paired-t test). Abbreviations: PREC, precontemplation; CON, contemplation; DET, determination; ACT, action; MAIN, maintenance; DIS, discrepancy; IMP, importance; S-EFF, self-efficacy; TEMP, temptation; READY, readiness-to-change; STAB, stabilization-of-change.

At baseline, several scores of the EMME−3 questionnaire—namely the stage of change scores—were significantly lower in the area of physical activity vs. healthy diet, with the notable exclusion of the maintenance score, and the temptation score was also lower (Figure 1). This suggests a much lower overall interest for physical activity in the participants.

**Figure 2:**
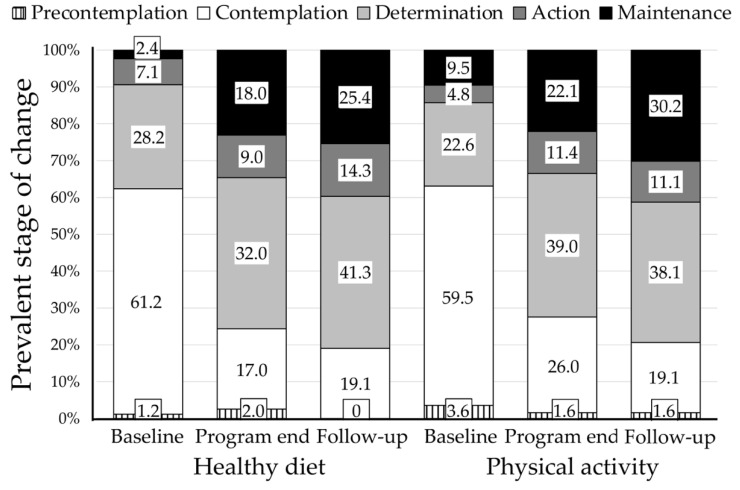
Prevalent stage of change at baseline and during the whole study period. The analysis covers the patients at enrollment (*n* = 86), at the end of the motivational interviewing (MI) program (*n* = 73) and at 6-month follow-up (*n* = 65). Note the progressive shift from the contemplation stage to determination, action and maintenance.

The prevalent stage of change was contemplation (approximately 60% in both areas) (Figure 2), with a larger prevalence of subjects in the precontemplation stage for physical activity compared to that for diet, but also the prevalence of maintenance was larger for physical activity. All scores varied considerably in response to treatment, and similarly in the two areas (Figure 2). The scores of precontemplation and contemplation significantly decreased, with a remarkable increase in action and maintenance, a reduction of discrepancy and temptation, an increase in self-efficacy, in readiness-to-change and stabilization-of change (not reported in detail). Most of these changes were maintained, and sometimes improved further, at the 6-month follow-up. The prevalent stage of change shifted from determination to action and maintenance, although a few cases in precontemplation did not move along the stages. By the end of follow up, the prevalence of patients in either precontemplation or contemplation moved from over 60% to approximately 20%, whereas the sum of patients in the action or maintenance stages increased from 9.5% in healthy diet and 14.3% in physical activity to 39.7% and 41.3%, respectively.

Behavioral and Anthropometric Effects

Following the MI program, we recorded significant changes in calorie intake, total physical activity and time spent sedentarily, and finally in body weight and waist circumference (Table 2). Overall, body weight decreased by nearly 2.5 kg and waist circumference by 2 cm. In particular, body weight decreased by 5% or more in 7.3% of cases at the end of the MI program, whereas at 6-month follow-up weight loss exceeded 10% in 7.4% of cases and 5% in another 7.4%, but in a few cases weight losses up to 15% of the initial body weight were recorded. 

**Figure 3:**
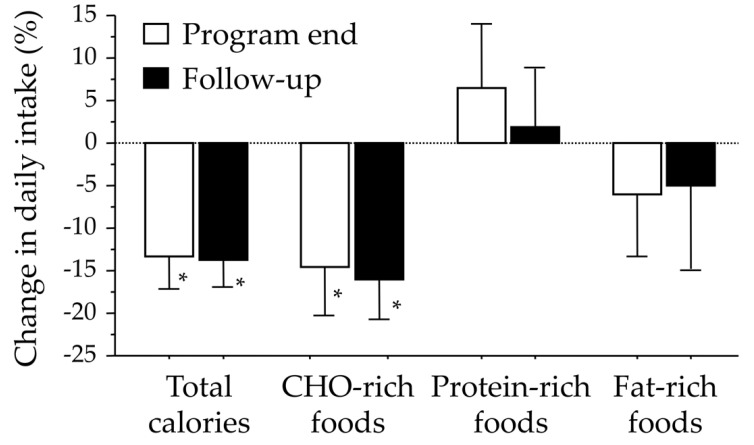
Percent changes in total calorie intake and in specific food items following participation in the group-adapted motivational interviewing program. * Denotes significant changes (one-sample ANOVA, *p* < 0.01).

Follow-up calorie intake averaged 1894 ± 596 kcal/day by the end-of-program evaluation, and this value was maintained at 6-month follow-up (1869 ± 614 kcal/day) (Figure 3). Among the different nutrients, we observed a marked reduction of carbohydrate-containing foods. The calories derived from carbohydrate-rich sources were reduced by 18.6% at the end of the program (*p* < 0.001), both as an effect of reduced weekly consumption throughout the day (soft drinks, candies, chocolate) or as reduced consumption at meals (less frequent consumption of pasta, bread and cake) and as smaller portion sizes; the restriction was maintained at follow-up ( −11.4%; *p* vs. baseline, <0.001). There was a small, non-significant increase in protein-containing food products, and a non-significant decrease in fat-rich foods ( −5 to −6%).

The amount of physical activity continued to increase throughout the study, to a median value of 17.0 [interquartile range, 30.2] MET-h/week at follow-up (*p* vs. end-of-program, 0.021) whereas the time spent sedentary was progressively reduced from 425 ±181 min/day to 353 ±138 and 346 ±145 by program end and follow-up, respectively (both, *p* <0.01).

## 4. Discussion

The study shows that an MI program is able to restart a virtuous circle along the five-stage cycle of change of Prochaska and Di Clemente [26]. This translates into healthy behavioral changes, characterized by decreased calorie intake—with appropriate food selection and specific reduction of carbohydrate-rich foods—higher physical activity and reduced sedentariness, and a new start toward obesity control.

In line with the spirit of MI, motivational groups aim to guide members to recognize what changes can improve their quality of life, what barriers they will encounter and what strengths can help overcome obstacles. This is based on theoretical models as well as on the use of strategies and communication skills, aimed at overcoming ambivalence and increasing patients’ willingness for changes [4]. The rationale of adding MI to traditional behavioral weight loss programs is solid and is very likely to improve the final results. By addressing ambivalence, MI is expected to tailor treatment to patients’ characteristics, to cope with resistance and to identify acceptable and realistic goals in a better way and using a distinctive therapeutic style and well-defined clinical procedures, not necessarily present in the routine practice of CBT [12]. MI also tends to smoothen the rather rigid classification of the different stages of changes as derived from Prochaska and Di Clemente [14], thus favoring a progressive change.

Addressing the problem of excess weight and change in a group context has several potential advantages: the group is supportive for the person in his/her willingness for action; confronting with people sharing similar life habits and problems makes one feel less alone; listening to successful experiences reported by peers can instill greater desire and hope and reinforce the sense of self-efficacy. This translates into a larger weight loss [27,28] and reduces dropouts [27], as also suggested by Italian guidelines [29]. However, working with a group rather than with individuals requires different strategies. By a specific communication style (open questions, affirmations, reflections, summaries) the therapist aims to elicit a change talk from participants, i.e., a language oriented to change during individual MI sessions, but an MI group cannot be reduced to individual MI mini sessions. In successful MI groups, the group itself develops into a positive social network in which members participate, influence and are influenced each other [10]. In order to promote this, the therapist makes use of strategies such as linking members, finding areas of common interest, and promoting their involvement in other members’ personal exploration and goal setting, finally supporting each other. Group members develop a sense of group identity that increases both their investment in the group and their openness to share personal thoughts with other members. By the end of the program, group members more clearly identify their needs and new skills and strategies to cope with, and also greater confidence to be successful.

The development of techniques favoring the interaction of all members may be jeopardized by different cultural background, interests and perspectives of participants and a careful selection is mandatory. Moreover, in our setting, a few group members were in a precontemplation stage at entry and remained totally unresponsive to treatment. A few more did not complete the program or were lost to follow-up. Attrition rates are very high in obesity treatment, irrespective of the type of treatment, and even higher in countries where treatment may be dispensed by a National health system, as occurs in Italy, with patients paying only a modest co-participation for visits or group therapy [30]. The 6-month attrition rates of 24% observed in the present program compares favorably with previous experiences of obesity treatment in Italy, where attrition rates were higher and highly related to a decline of motivation in response to very challenging weight loss expectations [1,31].

The MI program followed a previous psycho-educational or cognitive-behavioral treatment. In a systematic review and meta-analysis testing the effects of motivational interviewing associated with behavior therapy, Barrett et al. confirmed that the combined intervention was effective in promoting significant health outcomes [7]. We tested the effects of the MI program on both calorie intake and physical activity, the lifestyle mediators of overweight and obesity, and both areas improved. At baseline calorie intake was much higher than the amount suggested to reduce body weight during behavior therapy [32], and remained relatively higher-than-needed but stable, also following MI group therapy. On the contrary, the program had a very systematic effect on physical activity and sedentariness, more than doubling the amount of MET at follow-up. Strategies were suggested to increase physical activity during behavior therapy [33], but the effect of the MI program was much larger, probably resolving ambivalence in participants [34], as well as favoring the identification of barriers and the strategies to overcome them with the help of the group leader and members.

While individual MI has a large literature of well-designed clinical trials that support its efficacy, the usefulness of MI group programs was more recently reported, and the research mostly focused on substance use as a target behavior. Despite the small number of controlled studies, there is evidence that MI groups may also improve the recognition of ambivalence [35], increase self-efficacy, behavioral intentions and readiness to change [36,37], as well as increase treatment engagement and participation [38]. All these effects are similar to those reported for individual MI [39] and are consistent with our findings, where the virtuous circle along the five-stage Prochaska cycle was associated with increased scores of self-efficacy, readiness-to-change and stabilization-of change.

The study has both strengths and limitations. The strengths come from its well-defined methodology, careful selection of participants, extensive measures of psychological profile and outcomes. The limitations are the short-term assessment of outcomes and the need for a team leader well-trained in the strategies for conducting MI in a group setting. Between end-of-program and follow-up, most domains of the EMME−3 questionnaire remained stable, but they showed a modest decline in a few individuals. This was observed in the healthy diet area, where decreased action, readiness-to-change and stabilization-of-change were measured. This limitation adds to the attrition rate, increasing from 15% to 24% in an approximately 4-month period and requires a lot of attention.

The need for a team leader expert in MI with the ability to translate MI from face-to-face contact to group setting is another important limit. MI training has been extensively investigated in recent literature [40], considering that the final outcomes are definitely dependent on the team leader [41]. The group format of MI requires specific competence and additional tasks and the activities are far more complex to conduct than in individual programs [42]. During group meetings, there is no possibility to explore participants’ situation in depth, as is done during individual counseling to favor engagement. Paying too much attention to single participants would turn group sessions into individual mini-sessions, with other participants as observers, thus losing the opportunities offered by group treatment [43]. In an extensive review of MI for weight loss in primary care, Barnes and Ivezaj found that interventions were usually provided by mixed teams, including clinicians of various backgrounds (dietitians, nurses, medical doctors, exercise specialists or simply “trained non-specialists”) [44]. Group research has identified specific strategies (engagement in group process [45], group cohesiveness and mutual task involvement [46]) as key contributors to success [47]. This requires that a leader is trained in both MI and group therapy [48]. In our setting, MI intervention was carried out by a single, trained person, with an extensive experience of MI in the area of both obesity and other non-communicable disease [49,50], thus guaranteeing a non-eclectic course.

In summary, this report provides evidence that MI programs adapted for groups may be used also in a public health setting to stop relapse in individuals following a behavioral intervention for weight loss. The group format is likely to increase dissemination of the procedure, to intercept a large number of cases at risk, as well as to reduce costs. Long-term studies are needed to define the strategies to obtain the highest advantage by the intervention and whether additional meetings to reinforce motivation may strengthen the results.

## Figures and Tables

**Table 1:** Sociodemographic, clinical and behavioral characteristics of patients enrolled in the study.

Variable	All Cases (*n* = 86)	Men (*n* = 18)	Women (*n* = 68)	*p* Value
Age (years)	56.6 ± 9.2	60.4 ± 8.3	55.7 ± 9.2	0.076
Age at first diet (years)	28.8 ± 16.9	40.7 ± 12.1	26.4 ± 16.2	0.033
Education (secondary/high-school/degree) (%)	16.0/66.0/18.0	9.1/72.7/18.2	17.9/64.1/17.9	0.214
Job placement (housewife/employed/retired) (%)	7.8/56.9/35.3	0/58.3/41.7	10.3/56.4/33.3	0.585
Civil status (single/married/widowed (%)	27.5/70.6/1.9	18.2/81.8/0	30.0/67.5/2.5	0.617
Body weight (kg)	95.3 ± 19.8	108.0 ± 15.9	91.9 ± 19.4	0.002
Body weight at age 20 (kg)	62.3 ± 15.1	73.8 ± 17.9	59.6 ± 13.2	0.010
Lifetime maximum weight (kg)	97.7 ± 23.0	114.9 ± 19.3	93.6 ± 23.0	0.021
Body mass index (kg/m^2^)	34.8 ± 5.8	35.1 ± 4.2	34.8 ± 6.2	0.761
Obesity class (overweight/obesity I/obesity II/obesity III) (%)	22/32/34/12	13/27/60/0	24/33/27/16	0.143
Waist circumference (cm)	108.4 ± 12.3	116.3 ± 12.0	106.3 ± 11.6	0.003
Food intake (kcal/day)	2206 ± 765	2240 ± 639	2196 ± 803	0.869
- Calories from CHO-rich foods (%)	1400 ± 643	1464 ± 644	1383 ± 650	0.714
- Calories from protein-rich foods (%)	238 ± 100	235 ± 91	239 ± 103	0.917
- Calories from lipid-rich sources (%)	428 ± 174	414 ± 133	422 ± 185	0.762
Physical activity (MET-h/week) *	8.0 (10.5)	7.0 (13.5)	8.0 (9.9)	0.690
Sedentary time (min/day)	425 ± 181	523 ± 220	388 ± 170	0.034

* Median (interquartile range); *p* value (Mann–Whitney test). Calories from different food sources are calculated by grouping the different items of the food questionnaire in relation to their prevalent nutrient composition (cereals, bread, pasta, sweets, cakes/ice creams, potatoes and fruits for carbohydrates (CHO); meat for protein; pork meat, oil and cheese for lipids. The extra calorie intake associated with restaurant meals included in the questionnaire was not considered.

**Table 2:** Changes in behavioral and anthropometric parameters in response to the motivational interviewing program.

Variable	Δ Program End	*p* Value *	Δ 6-Month Follow-Up	*p* Value *
Calorie intake (kcal/day)	−400 ± 94	0.001	−370 ± 78	<0.001
Physical activity (MET-h/week)	+3.5 (11.4)	0.004	+7.5 (17.1)	<0.001
Sedentary time (min/day)	−60.0 (142)	0.005	−60 (175)	0.002
Body weight (kg)	−1.59 ± 0.30	<0.001	−2.45 ± 0.68	<0.001
Waist circumference (cm)	−1.48 ± 0.61	<0.001	−2.00 ± 0.67	0.026

Data are presented as mean ± SD or as median (interquartile range). Δ, Change in variable. * Paired t or Wilcoxon rank test.

## Data Availability

The data presented in this study are available on request from the corresponding author in aggregate form. The data are not publicly available due to privacy restrictions.
